# 1,3-Bis(2-quinolylcarbon­yl)-1*H*,3*H*-2,1,3-benzothia­diazole 2-oxide

**DOI:** 10.1107/S1600536809033182

**Published:** 2009-08-22

**Authors:** Lin-Hai Jing

**Affiliations:** a School of Chemistry and Chemical Engineering, China West Normal University, Nanchong 637002, People’s Republic of China

## Abstract

In the title compound, C_26_H_16_N_4_O_3_S, the thia­diazole ring adopts an envelope conformation, with the S atom occupying the flap position. The dihedral angle between the two quinoline ring systems is 55.32 (8)°. In the crystal, the mol­ecules are linked into chains along [010] by C—H⋯O hydrogen bonds. The chains are connected *via* π–π inter­actions involving one of the pyridine rings [centroid–centroid distance = 3.5558 (18) Å].

## Related literature

For benzothia­diazole derivatives as potential anti­depressants, see: Pullar *et al.* (2000 ▶).
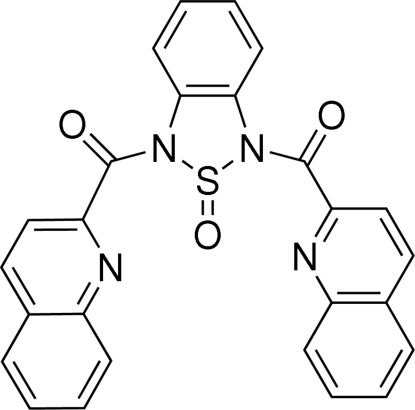


## Experimental

### 

#### Crystal data

C_26_H_16_N_4_O_3_S
*M* *_r_* = 464.49
Triclinic, 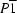

*a* = 8.0914 (5) Å
*b* = 10.2920 (6) Å
*c* = 12.8843 (7) Å
α = 93.232 (2)°
β = 93.791 (2)°
γ = 101.746 (2)°
*V* = 1045.51 (11) Å^3^
*Z* = 2
Mo *K*α radiation
μ = 0.20 mm^−1^
*T* = 153 K
0.26 × 0.18 × 0.12 mm

#### Data collection

Rigaku R-AXIS RAPID diffractometer
Absorption correction: none
10347 measured reflections
4764 independent reflections
3074 reflections with *I* > 2σ(*I*)
*R*_int_ = 0.059

#### Refinement

*R*[*F* ^2^ > 2σ(*F* ^2^)] = 0.055
*wR*(*F* ^2^) = 0.189
*S* = 1.01
4764 reflections
307 parameters
H-atom parameters constrained
Δρ_max_ = 0.41 e Å^−3^
Δρ_min_ = −0.66 e Å^−3^

### 

Data collection: *RAPID-AUTO* (Rigaku, 2004 ▶); cell refinement: *RAPID-AUTO*; data reduction: *RAPID-AUTO*; program(s) used to solve structure: *SHELXS97* (Sheldrick, 2008 ▶); program(s) used to refine structure: *SHELXL97* (Sheldrick, 2008 ▶); molecular graphics: *XP* in *SHELXTL* (Sheldrick, 2008 ▶); software used to prepare material for publication: *SHELXL97*.

## Supplementary Material

Crystal structure: contains datablocks global, I. DOI: 10.1107/S1600536809033182/ci2890sup1.cif

Structure factors: contains datablocks I. DOI: 10.1107/S1600536809033182/ci2890Isup2.hkl

Additional supplementary materials:  crystallographic information; 3D view; checkCIF report

## Figures and Tables

**Table 1:** Hydrogen-bond geometry (Å, °)

*D*—H⋯*A*	*D*—H	H⋯*A*	*D*⋯*A*	*D*—H⋯*A*
C13—H13⋯O2^i^	0.95	2.51	3.262 (4)	136

Symmetry code: (i) 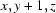.

**Fig. 1.**
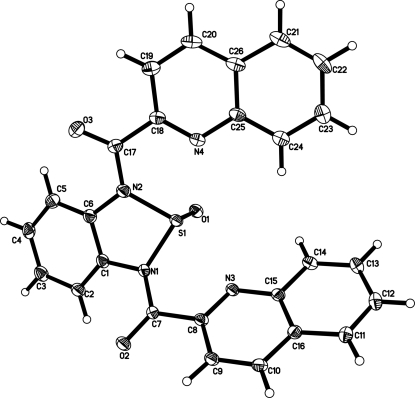
The molecular structure of the title compound, showing 30% probability displacement ellipsoids and the atomic numbering.

## supplementary crystallographic information

### Comment

Some of the benzothiadiazole derivatives are important and potential antidepressants (Pullar *et al.*, 2000). Here, we report the crystal structure of the title compound (Fig.1).

Bond lengths and angles are normal. The N3- and N4-quinoline ring systems are planar, with maximum deviations of 0.022 (3) Å and 0.018 (3) Å, respectively, for atoms C16 and N4. The five-membered ring (N1/N2/C1/C6/S1) adopts an envelope conformation.

The crystal packing is stabilized by C—H···O hydrogen bonds (Table 1) which link the molecules to form a chain along the *b* axis.

### Experimental

Quinoline-2-carboxylic acid (2 mmol) and an excess of thionyl chloride (6 mmol) were reacted at 333 K for 6 h. The solution was distilled under reduced pressure and a bright yellow solid was obtained. *O*-Phenylenediamine (1 mmol) in tetrahydrofuran (20 ml) was added to the bright yellow solid and reacted at 333 K for 6 h. Another excess of thionyl chloride (6 mmol) was added to the reaction system and the reaction was continued under reflux for 6 h.The reaction system was then cooled to ambient temperature and filtered to remove the tetrahydrofuran and unreacted thionyl chloride. The precipitate was dissolved in dimethylformamide and allowed to stand for one month at ambient temperature, after which time white single crystals of the title compound suitable for X-ray diffraction were obtained.

### Refinement

H atoms were placed in calculated positions, with C-H = 0.95 Å, and refined using a riding model, with *U*_iso_(H) = 1.2*U*_eq_(C).

### Crystal data

**Table:**

C_26_H_16_N_4_O_3_S	*Z* = 2
*M**_r_* = 464.49	*F*(000) = 480
Triclinic, *P*1	*D*_x_ = 1.475 Mg m^−^^3^
Hall symbol: -P 1	Mo *K*α radiation, λ = 0.71073 Å
*a* = 8.0914 (5) Å	Cell parameters from 6929 reflections
*b* = 10.2920 (6) Å	θ = 3.1–27.5°
*c* = 12.8843 (7) Å	µ = 0.20 mm^−^^1^
α = 93.232 (2)°	*T* = 153 K
β = 93.791 (2)°	Block, white
γ = 101.746 (2)°	0.26 × 0.18 × 0.12 mm
*V* = 1045.51 (11) Å^3^	

### Data collection

**Table:**

Rigaku R-AXIS RAPID diffractometer	3074 reflections with *I* > 2σ(*I*)
Radiation source: fine-focus sealed tube	*R*_int_ = 0.059
graphite	θ_max_ = 27.5°, θ_min_ = 3.1°
ω scans	*h* = −10→10
10347 measured reflections	*k* = −13→13
4764 independent reflections	*l* = −16→16

### Refinement

**Table:**

Refinement on *F*^2^	Primary atom site location: structure-invariant direct methods
Least-squares matrix: full	Secondary atom site location: difference Fourier map
*R*[*F*^2^ > 2σ(*F*^2^)] = 0.055	Hydrogen site location: inferred from neighbouring sites
*wR*(*F*^2^) = 0.189	H-atom parameters constrained
*S* = 1.01	*w* = 1/[σ^2^(*F*_o_^2^) + (0.1*P*)^2^ + 0.398*P*] where *P* = (*F*_o_^2^ + 2*F*_c_^2^)/3
4764 reflections	(Δ/σ)_max_ = 0.001
307 parameters	Δρ_max_ = 0.41 e Å^−^^3^
0 restraints	Δρ_min_ = −0.66 e Å^−^^3^

### Special details

**Table:**

Geometry. All e.s.d.'s (except the e.s.d. in the dihedral angle between two l.s. planes) are estimated using the full covariance matrix. The cell e.s.d.'s are taken into account individually in the estimation of e.s.d.'s in distances, angles and torsion angles; correlations between e.s.d.'s in cell parameters are only used when they are defined by crystal symmetry. An approximate (isotropic) treatment of cell e.s.d.'s is used for estimating e.s.d.'s involving l.s. planes.
Refinement. Refinement of *F*^2^ against ALL reflections. The weighted *R*-factor *wR* and goodness of fit *S* are based on *F*^2^, conventional *R*-factors *R* are based on *F*, with *F* set to zero for negative *F*^2^. The threshold expression of *F*^2^ > σ(*F*^2^) is used only for calculating *R*-factors(gt) *etc*. and is not relevant to the choice of reflections for refinement. *R*-factors based on *F*^2^ are statistically about twice as large as those based on *F*, and *R*- factors based on ALL data will be even larger.

### Fractional atomic coordinates and isotropic or equivalent isotropic displacement parameters (Å^2^)

**Table:**

	*x*	*y*	*z*	*U*_iso_*/*U*_eq_	
S1	0.69178 (9)	0.31618 (7)	0.73387 (5)	0.0259 (2)	
N1	0.6436 (3)	0.1832 (2)	0.81493 (19)	0.0270 (5)	
N2	0.7768 (3)	0.2062 (2)	0.6523 (2)	0.0307 (6)	
N3	0.4869 (3)	0.3845 (2)	0.86504 (18)	0.0247 (5)	
N4	0.7299 (3)	0.4345 (2)	0.56732 (19)	0.0298 (6)	
O1	0.8377 (2)	0.40615 (19)	0.78560 (15)	0.0294 (5)	
O2	0.4360 (3)	0.0432 (2)	0.89052 (18)	0.0361 (5)	
O3	0.8451 (3)	0.1354 (2)	0.49310 (17)	0.0419 (6)	
C1	0.7478 (4)	0.0898 (3)	0.8014 (2)	0.0286 (6)	
C2	0.7825 (4)	−0.0001 (3)	0.8718 (2)	0.0323 (7)	
H2	0.7318	−0.0064	0.9362	0.039*	
C3	0.8936 (4)	−0.0807 (3)	0.8452 (3)	0.0358 (7)	
H3	0.9189	−0.1434	0.8919	0.043*	
C4	0.9685 (4)	−0.0710 (3)	0.7510 (3)	0.0349 (7)	
H4	1.0437	−0.1274	0.7342	0.042*	
C5	0.9345 (4)	0.0204 (3)	0.6810 (2)	0.0340 (7)	
H5	0.9867	0.0278	0.6171	0.041*	
C6	0.8234 (4)	0.0996 (3)	0.7071 (2)	0.0292 (6)	
C7	0.4910 (3)	0.1526 (3)	0.8607 (2)	0.0250 (6)	
C8	0.3985 (3)	0.2634 (3)	0.8743 (2)	0.0254 (6)	
C9	0.2277 (3)	0.2344 (3)	0.8967 (2)	0.0273 (6)	
H9	0.1702	0.1455	0.9033	0.033*	
C10	0.1467 (4)	0.3382 (3)	0.9088 (2)	0.0285 (6)	
H10	0.0309	0.3219	0.9232	0.034*	
C11	0.1604 (4)	0.5824 (3)	0.9083 (2)	0.0298 (6)	
H11	0.0446	0.5719	0.9218	0.036*	
C12	0.2545 (4)	0.7056 (3)	0.8968 (2)	0.0342 (7)	
H12	0.2034	0.7807	0.9033	0.041*	
C13	0.4257 (4)	0.7242 (3)	0.8756 (2)	0.0327 (7)	
H13	0.4882	0.8111	0.8671	0.039*	
C14	0.5021 (4)	0.6186 (3)	0.8672 (2)	0.0299 (6)	
H14	0.6184	0.6318	0.8543	0.036*	
C15	0.4078 (3)	0.4884 (3)	0.8777 (2)	0.0253 (6)	
C16	0.2359 (4)	0.4696 (3)	0.8999 (2)	0.0267 (6)	
C17	0.8056 (4)	0.2214 (3)	0.5479 (2)	0.0321 (7)	
C18	0.7883 (4)	0.3522 (3)	0.5066 (2)	0.0291 (6)	
C19	0.8409 (4)	0.3778 (3)	0.4052 (2)	0.0369 (7)	
H19	0.8819	0.3140	0.3636	0.044*	
C20	0.8294 (4)	0.5000 (4)	0.3707 (2)	0.0392 (8)	
H20	0.8667	0.5233	0.3045	0.047*	
C21	0.7451 (4)	0.7167 (3)	0.4011 (3)	0.0414 (8)	
H21	0.7778	0.7432	0.3347	0.050*	
C22	0.6821 (4)	0.8002 (3)	0.4645 (3)	0.0442 (9)	
H22	0.6720	0.8850	0.4425	0.053*	
C23	0.6310 (4)	0.7630 (3)	0.5634 (3)	0.0423 (8)	
H23	0.5855	0.8223	0.6070	0.051*	
C24	0.6469 (4)	0.6424 (3)	0.5963 (2)	0.0355 (7)	
H24	0.6134	0.6179	0.6629	0.043*	
C25	0.7126 (4)	0.5542 (3)	0.5320 (2)	0.0299 (6)	
C26	0.7633 (4)	0.5896 (3)	0.4320 (2)	0.0344 (7)	

### Atomic displacement parameters (Å^2^)

**Table:**

	*U*^11^	*U*^22^	*U*^33^	*U*^12^	*U*^13^	*U*^23^
S1	0.0268 (4)	0.0243 (4)	0.0281 (4)	0.0063 (3)	0.0061 (3)	0.0076 (3)
N1	0.0305 (13)	0.0221 (11)	0.0321 (12)	0.0105 (10)	0.0093 (10)	0.0068 (10)
N2	0.0303 (13)	0.0303 (13)	0.0349 (13)	0.0104 (11)	0.0086 (10)	0.0097 (11)
N3	0.0255 (12)	0.0234 (11)	0.0259 (11)	0.0055 (9)	0.0035 (9)	0.0060 (10)
N4	0.0307 (13)	0.0310 (13)	0.0278 (12)	0.0042 (11)	0.0046 (10)	0.0080 (11)
O1	0.0271 (10)	0.0271 (10)	0.0329 (11)	0.0024 (8)	0.0023 (8)	0.0043 (9)
O2	0.0350 (12)	0.0281 (11)	0.0502 (13)	0.0108 (9)	0.0157 (10)	0.0154 (10)
O3	0.0513 (15)	0.0421 (13)	0.0348 (12)	0.0150 (12)	0.0085 (10)	−0.0010 (11)
C1	0.0259 (14)	0.0245 (13)	0.0377 (16)	0.0083 (12)	0.0047 (12)	0.0066 (12)
C2	0.0326 (16)	0.0296 (15)	0.0371 (16)	0.0085 (13)	0.0073 (13)	0.0090 (13)
C3	0.0306 (16)	0.0295 (15)	0.0498 (19)	0.0100 (13)	0.0035 (14)	0.0105 (14)
C4	0.0290 (15)	0.0278 (15)	0.0503 (19)	0.0118 (13)	0.0043 (14)	0.0016 (14)
C5	0.0297 (16)	0.0356 (16)	0.0382 (16)	0.0093 (13)	0.0062 (13)	0.0026 (14)
C6	0.0277 (15)	0.0275 (14)	0.0345 (15)	0.0091 (12)	0.0053 (12)	0.0063 (13)
C7	0.0281 (14)	0.0216 (13)	0.0271 (13)	0.0076 (11)	0.0053 (11)	0.0045 (11)
C8	0.0264 (14)	0.0266 (14)	0.0242 (13)	0.0059 (11)	0.0031 (11)	0.0072 (12)
C9	0.0271 (14)	0.0262 (14)	0.0294 (14)	0.0051 (11)	0.0046 (11)	0.0075 (12)
C10	0.0251 (14)	0.0319 (15)	0.0294 (14)	0.0055 (12)	0.0057 (11)	0.0086 (12)
C11	0.0307 (15)	0.0294 (14)	0.0306 (14)	0.0102 (12)	0.0013 (12)	0.0006 (13)
C12	0.0387 (17)	0.0276 (15)	0.0387 (17)	0.0135 (13)	0.0001 (13)	0.0023 (13)
C13	0.0360 (16)	0.0255 (14)	0.0374 (16)	0.0078 (12)	0.0000 (13)	0.0071 (13)
C14	0.0299 (15)	0.0306 (15)	0.0286 (14)	0.0035 (12)	0.0014 (11)	0.0083 (13)
C15	0.0283 (14)	0.0266 (13)	0.0224 (13)	0.0075 (11)	0.0028 (11)	0.0065 (11)
C16	0.0287 (14)	0.0289 (14)	0.0238 (13)	0.0086 (12)	0.0026 (11)	0.0046 (12)
C17	0.0327 (16)	0.0346 (16)	0.0301 (15)	0.0079 (13)	0.0076 (12)	0.0031 (13)
C18	0.0268 (14)	0.0332 (15)	0.0250 (14)	0.0007 (12)	0.0018 (11)	0.0040 (13)
C19	0.0360 (17)	0.0472 (19)	0.0272 (15)	0.0071 (15)	0.0033 (13)	0.0041 (15)
C20	0.0341 (17)	0.055 (2)	0.0279 (15)	0.0038 (15)	0.0054 (13)	0.0157 (16)
C21	0.0404 (18)	0.0418 (18)	0.0382 (17)	−0.0024 (15)	−0.0037 (14)	0.0174 (16)
C22	0.0432 (19)	0.0295 (16)	0.055 (2)	−0.0025 (14)	−0.0107 (16)	0.0165 (17)
C23	0.0421 (19)	0.0340 (17)	0.051 (2)	0.0087 (15)	−0.0011 (15)	0.0038 (16)
C24	0.0359 (17)	0.0356 (16)	0.0342 (16)	0.0041 (14)	0.0030 (13)	0.0074 (14)
C25	0.0263 (14)	0.0319 (15)	0.0288 (14)	−0.0008 (12)	−0.0001 (11)	0.0075 (13)
C26	0.0283 (15)	0.0415 (17)	0.0300 (15)	−0.0020 (13)	−0.0010 (12)	0.0123 (14)

### Geometric parameters (Å, °)

**Table:**

S1—O1	1.441 (2)	C10—H10	0.95
S1—N1	1.764 (2)	C11—C12	1.361 (4)
S1—N2	1.771 (3)	C11—C16	1.420 (4)
N1—C7	1.390 (3)	C11—H11	0.95
N1—C1	1.413 (3)	C12—C13	1.407 (4)
N2—C17	1.393 (4)	C12—H12	0.95
N2—C6	1.437 (3)	C13—C14	1.359 (4)
N3—C8	1.321 (3)	C13—H13	0.95
N3—C15	1.361 (3)	C14—C15	1.419 (4)
N4—C18	1.300 (4)	C14—H14	0.95
N4—C25	1.368 (3)	C15—C16	1.416 (4)
O2—C7	1.217 (3)	C17—C18	1.504 (4)
O3—C17	1.208 (4)	C18—C19	1.422 (4)
C1—C2	1.388 (4)	C19—C20	1.376 (5)
C1—C6	1.396 (4)	C19—H19	0.95
C2—C3	1.388 (4)	C20—C26	1.391 (5)
C2—H2	0.95	C20—H20	0.95
C3—C4	1.391 (5)	C21—C22	1.349 (5)
C3—H3	0.95	C21—C26	1.422 (4)
C4—C5	1.393 (4)	C21—H21	0.95
C4—H4	0.95	C22—C23	1.415 (5)
C5—C6	1.376 (4)	C22—H22	0.95
C5—H5	0.95	C23—C24	1.362 (4)
C7—C8	1.495 (4)	C23—H23	0.95
C8—C9	1.406 (4)	C24—C25	1.400 (4)
C9—C10	1.369 (4)	C24—H24	0.95
C9—H9	0.95	C25—C26	1.422 (4)
C10—C16	1.412 (4)		
			
O1—S1—N1	105.89 (12)	C11—C12—H12	119.3
O1—S1—N2	104.62 (12)	C13—C12—H12	119.3
N1—S1—N2	86.41 (10)	C14—C13—C12	120.4 (3)
C7—N1—C1	122.2 (2)	C14—C13—H13	119.8
C7—N1—S1	122.68 (17)	C12—C13—H13	119.8
C1—N1—S1	112.25 (17)	C13—C14—C15	119.8 (3)
C17—N2—C6	122.0 (3)	C13—C14—H14	120.1
C17—N2—S1	125.42 (19)	C15—C14—H14	120.1
C6—N2—S1	112.54 (19)	N3—C15—C16	121.9 (2)
C8—N3—C15	118.0 (2)	N3—C15—C14	118.2 (2)
C18—N4—C25	118.8 (2)	C16—C15—C14	119.9 (2)
C2—C1—C6	121.0 (3)	C10—C16—C15	117.9 (2)
C2—C1—N1	126.8 (3)	C10—C16—C11	123.4 (3)
C6—C1—N1	112.2 (2)	C15—C16—C11	118.7 (3)
C3—C2—C1	118.0 (3)	O3—C17—N2	122.2 (3)
C3—C2—H2	121.0	O3—C17—C18	121.2 (3)
C1—C2—H2	121.0	N2—C17—C18	116.6 (3)
C2—C3—C4	120.9 (3)	N4—C18—C19	125.0 (3)
C2—C3—H3	119.5	N4—C18—C17	117.4 (2)
C4—C3—H3	119.5	C19—C18—C17	117.6 (3)
C3—C4—C5	120.9 (3)	C20—C19—C18	116.5 (3)
C3—C4—H4	119.6	C20—C19—H19	121.8
C5—C4—H4	119.6	C18—C19—H19	121.8
C6—C5—C4	118.2 (3)	C19—C20—C26	120.4 (3)
C6—C5—H5	120.9	C19—C20—H20	119.8
C4—C5—H5	120.9	C26—C20—H20	119.8
C5—C6—C1	121.1 (2)	C22—C21—C26	121.0 (3)
C5—C6—N2	128.8 (3)	C22—C21—H21	119.5
C1—C6—N2	110.0 (2)	C26—C21—H21	119.5
O2—C7—N1	122.7 (2)	C21—C22—C23	120.8 (3)
O2—C7—C8	121.1 (2)	C21—C22—H22	119.6
N1—C7—C8	116.2 (2)	C23—C22—H22	119.6
N3—C8—C9	124.4 (2)	C24—C23—C22	120.1 (3)
N3—C8—C7	116.1 (2)	C24—C23—H23	120.0
C9—C8—C7	119.5 (2)	C22—C23—H23	120.0
C10—C9—C8	118.0 (2)	C23—C24—C25	120.2 (3)
C10—C9—H9	121.0	C23—C24—H24	119.9
C8—C9—H9	121.0	C25—C24—H24	119.9
C9—C10—C16	119.8 (2)	N4—C25—C24	119.1 (2)
C9—C10—H10	120.1	N4—C25—C26	120.4 (3)
C16—C10—H10	120.1	C24—C25—C26	120.4 (3)
C12—C11—C16	119.8 (3)	C20—C26—C21	123.6 (3)
C12—C11—H11	120.1	C20—C26—C25	118.9 (3)
C16—C11—H11	120.1	C21—C26—C25	117.5 (3)
C11—C12—C13	121.4 (3)		
			
O1—S1—N1—C7	118.1 (2)	C12—C13—C14—C15	1.3 (5)
N2—S1—N1—C7	−137.7 (3)	C8—N3—C15—C16	−0.1 (4)
O1—S1—N1—C1	−80.7 (2)	C8—N3—C15—C14	−179.6 (3)
N2—S1—N1—C1	23.5 (2)	C13—C14—C15—N3	177.7 (3)
O1—S1—N2—C17	−93.9 (3)	C13—C14—C15—C16	−1.9 (4)
N1—S1—N2—C17	160.6 (3)	C9—C10—C16—C15	0.5 (4)
O1—S1—N2—C6	83.0 (2)	C9—C10—C16—C11	178.1 (3)
N1—S1—N2—C6	−22.5 (2)	N3—C15—C16—C10	0.0 (4)
C7—N1—C1—C2	−40.6 (5)	C14—C15—C16—C10	179.5 (3)
S1—N1—C1—C2	158.0 (3)	N3—C15—C16—C11	−177.7 (3)
C7—N1—C1—C6	142.0 (3)	C14—C15—C16—C11	1.8 (4)
S1—N1—C1—C6	−19.3 (3)	C12—C11—C16—C10	−178.8 (3)
C6—C1—C2—C3	−0.5 (5)	C12—C11—C16—C15	−1.2 (4)
N1—C1—C2—C3	−177.7 (3)	C6—N2—C17—O3	12.3 (5)
C1—C2—C3—C4	0.3 (5)	S1—N2—C17—O3	−171.1 (2)
C2—C3—C4—C5	0.3 (5)	C6—N2—C17—C18	−166.5 (3)
C3—C4—C5—C6	−0.8 (5)	S1—N2—C17—C18	10.1 (4)
C4—C5—C6—C1	0.6 (5)	C25—N4—C18—C19	1.7 (5)
C4—C5—C6—N2	175.4 (3)	C25—N4—C18—C17	−180.0 (3)
C2—C1—C6—C5	0.1 (5)	O3—C17—C18—N4	173.6 (3)
N1—C1—C6—C5	177.6 (3)	N2—C17—C18—N4	−7.6 (4)
C2—C1—C6—N2	−175.6 (3)	O3—C17—C18—C19	−7.8 (5)
N1—C1—C6—N2	1.9 (4)	N2—C17—C18—C19	171.0 (3)
C17—N2—C6—C5	18.0 (5)	N4—C18—C19—C20	0.6 (5)
S1—N2—C6—C5	−159.0 (3)	C17—C18—C19—C20	−177.8 (3)
C17—N2—C6—C1	−166.8 (3)	C18—C19—C20—C26	−2.2 (5)
S1—N2—C6—C1	16.2 (3)	C26—C21—C22—C23	−0.6 (5)
C1—N1—C7—O2	−1.1 (5)	C21—C22—C23—C24	0.8 (5)
S1—N1—C7—O2	158.3 (2)	C22—C23—C24—C25	−0.5 (5)
C1—N1—C7—C8	177.2 (3)	C18—N4—C25—C24	178.9 (3)
S1—N1—C7—C8	−23.4 (4)	C18—N4—C25—C26	−2.2 (4)
C15—N3—C8—C9	−0.2 (4)	C23—C24—C25—N4	179.0 (3)
C15—N3—C8—C7	−179.5 (2)	C23—C24—C25—C26	0.0 (5)
O2—C7—C8—N3	162.9 (3)	C19—C20—C26—C21	−179.1 (3)
N1—C7—C8—N3	−15.4 (4)	C19—C20—C26—C25	1.7 (5)
O2—C7—C8—C9	−16.4 (4)	C22—C21—C26—C20	−179.1 (3)
N1—C7—C8—C9	165.2 (3)	C22—C21—C26—C25	0.1 (5)
N3—C8—C9—C10	0.7 (4)	N4—C25—C26—C20	0.5 (5)
C7—C8—C9—C10	179.9 (3)	C24—C25—C26—C20	179.5 (3)
C8—C9—C10—C16	−0.8 (4)	N4—C25—C26—C21	−178.7 (3)
C16—C11—C12—C13	0.7 (5)	C24—C25—C26—C21	0.2 (4)
C11—C12—C13—C14	−0.8 (5)		

### Hydrogen-bond geometry (Å, °)

**Table:**

*D*—H···*A*	*D*—H	H···*A*	*D*···*A*	*D*—H···*A*
C13—H13···O2^i^	0.95	2.51	3.262 (4)	136

Symmetry codes: (i) *x*, *y*+1, *z*.
